# To grunt or not to grunt: Factors governing call production in female olive baboons, *Papio anubis*

**DOI:** 10.1371/journal.pone.0204601

**Published:** 2018-11-02

**Authors:** Joan B. Silk, Eila R. Roberts, Veronika Städele, Shirley C. Strum

**Affiliations:** 1 School of Human Evolution and Social Change, Arizona State University, Tempe, Arizona, United States of America; 2 Institute for Human Origins, Arizona State University, Tempe, Arizona, United States of America; 3 Department of Primatology, Max Planck Institute for Evolutionary Anthropology, Leipzig, Germany; 4 Department of Anthropology, University of California San Diego, San Diego, California, United States of America; 5 Uaso Ngiro Baboon Project, Nairobi, Kenya; 6 Kenya Wildlife Service, Nairobi, Kenya; 7 African Conservation Centre, Nairobi, Kenya; University of York, UNITED KINGDOM

## Abstract

Vocal signals often play an important role in synchronizing the activities of group members, coordinating decisions about when and where to travel, and facilitating social interactions in which there are potential conflicts of interest. Here, we show that when female olive baboons (*Papio anubis*) give low amplitude grunts after approaching other females, they are less likely to behave aggressively toward their partners and more likely to handle their partners’ infants and interact affiliatively with them. In addition, females are more likely to grunt after they approach lower ranking females than after they approach higher ranking females and are less likely to grunt after they approach their own mothers and daughters than after they approach other females. These patterns, which are strikingly similar to patterns previously reported in chacma baboons (*Papio ursinus)* support the hypothesis that grunts function as signals of benign intent. Moreover, they suggest that actors’ decisions about whether to grunt or remain silent are influenced by the social context, particularly their partners’ likely response to their approach. Taken together, the patterning of grunts in olive and chacma baboon suggests that these vocalizations play an important in reducing uncertainty about actors’ intentions and facilitate nonaggressive social interactions.

## Introduction

In mammals, vocal signals can encode information about the caller’s identity, sex, genetic quality, dominance status, and emotional state [1–8], and vocalizations often play an important role in synchronizing the activities and movements of group members, maintaining contact between widely dispersed individuals, and reducing uncertainty about the outcome of social interactions [9–13]. For example, African elephants (*Loxodonta africana*) use low-frequency harmonically-rich rumbles to maintain contact with distant members of their social networks [11]. These calls contain acoustic information about individual sex, size, identity, and emotional state [14,15]. Northern elephant seals (*Mirounga angustirostris*) recognize the rhythm and timbre of other males’ threat vocalizations, and use this information to adjust their behavioral responses [16]. Meerkats (*Suricata suricatta*) give moving calls before they start to move, and group moves are more likely to occur when at least three individuals join in a calling chorus [17]. Female rhesus macaques (*Macaca mulatta*) give contact calls (grunts, girneys, coos) as they approach other members of their groups, and these calls are reliable signals that the callers will not behave aggressively [18]. Thus, vocal signals are a rich source of information for animals in social groups.

Playback studies have revealed “the exquisite skills of receivers, who take in and weigh information from different sources, to make adaptive decisions” ([19] page 76). Listeners’ responses to calls are influenced by a variety of factors, including their knowledge of the caller’s identity, the type of call that is given, and the history of their recent interactions with the caller [13]. For example, when female vervet monkeys (*Chlorocebus pygerythrus*) hear the scream of an infant, they look toward the infant’s mother [20].

Less is known about the factors that influence the production and usage of calls, partly because it is more difficult to experimentally manipulate call production than call perception. However, in an experiment in which chimpanzees were exposed to a snake model, chimpanzees were more likely to give alarm calls in the presence of individuals who were aware that the snake was present than in the presence of individuals who were unaware of the snake’s presence [21]. Female Japanese macaques (*Macaca fuscata*) are more likely to exchange contact calls (coos) with the females that they groom most often than with other females [22]. Nonetheless, our knowledge of the factors that influence call usage in most taxa remains limited.

There have been a number of studies of the acoustic structure, function, and usage of contact calls (grunts) by chacma baboons (*Papio ursinus*). Grunts are low amplitude, harmonically rich calls [23] which are most often given as the group begins to move through an open area [24, 25] and in social contexts [25–31]. Grunts are individually identifiable [23,32] and listeners seem to be able to determine when they are the target of others’ grunts [33]. As in rhesus macaques [18], grunts given in social contexts seem to function as signals of benign intent and facilitate nonaggressive social interactions, such as infant handling [28,31]. Grunts are also effective in reconciling conflicts with former opponents [26–28] and their close kin [29]. Analyses of naturally occurring sequences of interactions show that female chacma baboons take a variety of contextual factors into account when they decide whether to grunt or remain silent after approaching other females [34]. Females are more likely to grunt as they approach lower ranking females whom they might intimidate, threaten, or attack, than as they approach higher ranking females, whom they are unlikely to harass. In addition, females are less likely to grunt as they approach their own mothers and daughters, with whom they have highly affiliative and nonaggressive relationships, than they are to grunt as they approach other females. These data suggest that grunts reduce uncertainty about the likely outcome of an interaction between pairs of females whose relationship is not consistently affiliative.

Call structure is strongly influenced by phylogeny, but the usage of calls may differ among closely related species [35]. For example, the call repertoire of guinea baboons (*Papio papio*) is identical to the call repertoire of chacma baboons, but there are a number of differences in the ways that calls are used [36,37]. Male chacma baboons often emit loud calls in agonistic conflicts with other males and these calls provide reliable information about male fighting ability [38,39], while male guinea baboons (*Papio papio*) rarely use long calls in this context [37]. Female chacma and yellow baboons (*Papio cynocephalus*) routinely vocalize after copulating, and these calls are thought to incite male-male competition [40], but female guinea baboons rarely call after copulating [37]. These differences in the usage of vocalizations may reflect species-specific differences in social organization and reproductive strategies, as male guinea baboons form stronger social bonds and compete less vigorously for access to females than male chacma baboons do [37]. Studies closely related species can provide valuable insights about the selective factors that underly variation in call production and usage.

Here, we examine the factors that influence the function and usage of grunts by female olive baboons. There are a number of similarities in the social organization and behavior of olive and chacma baboons [41,42]. For example, both species form multi-male, multi-female groups from which males disperse at puberty. Like female chacma baboons, olive baboons form their strongest bonds with their own mothers and mature daughters and establish linear matrilineal dominance hierarchies. Females in both species are attracted to newborn infants and seem strongly motivated to handle them [30,43,44]. These similarities provide an important opportunity to determine whether the patterns observed in chacma baboons are robust. Based on the similarities in social organization between olive and chacma baboons, we predicted that olive baboons would use grunts in much the same way that chacma baboons do. Thus, we predicted that grunts would function as signals of benign intent in olive baboons, and would be associated with a higher probability that peaceful interactions would occur and a lower probability that aggression would occur. We also expected females to grunt to mothers of young infants more than they grunted to other females. We predicted that grunts would be more likely to be given when the outcome of approaches was uncertain. Thus, females would grunt more to lower ranking females than to higher ranking females, and less to their own mothers and daughters than to other females. We also tested a novel prediction that proximity bouts that began with grunts would last longer than proximity bouts that did not begin with grunts. Finally, we extended the analysis to consider how grunts affected the likelihood that recipients of approaches would initiate aggression, infant handling, and affiliation toward females that approached them.

## Results

### Study site

This study is based on observations of females in two groups of olive baboons that range in Mukogodo region of Laikipia North on the Laikipia Plateau of central Kenya. These groups are part of a larger population that is monitored by the Uaso Ngiro Baboon Project (UNBP), directed by Dr. Shirley C. Strum. The females in the groups that we studied are mainly descendants of Pumphouse Gang (PHG), one of two groups that were successfully translocated from Kekopey (Gilgil), Kenya, to Laikipia in 1984 [45]. PHG fissioned in a process that lasted from 2009 to 2011, producing two daughter groups. The larger of the two daughter groups retained the original name, PHG, and the smaller group was named Enkai (ENK). PHG fissioned again in a process that lasted from 2010 and was complete in July of 2013 and the larger daughter group retained the original group name. Our study focused these two groups, which occupied overlapping home ranges during our study.

The study groups range in a dry savanna habitat that included grassy plains, acacia woodlands, and thin riverine forests located on the banks of sandy riverbeds. Rainfall is typically concentrated during two wet seasons (March-June, November-December), but droughts have become increasingly common [46]. The baboons feed on a variety of grasses, herbs, sedges, and the flowers and the fruits and pods of a variety of shrubs and trees including several *Acacia* species. Recently (*Opuntia stricta*), a non-indigenous cactus, has invaded the area [46], and has become an important part of the diet.

### Subjects

We conducted focal samples on all of the adult and sub-adult females in the study groups. Females are considered to be sub-adults after they begin their first sexual swellings, and adult when they produce their first infants. The focal females ranged in age from 3.62 to 25.24 years of age. The exact age of one adult female was not known because she immigrated from a local troop as a juvenile.

### Data collection

We conducted 15-minute focal observations on all adult and sub-adult females in the study groups. Observations during morning hours (0600–1200) were conducted six days a week. During focal samples, observers recorded approaches by adults and sub-adults to within 1 meter of the focal animal and moves to more than 1 meter away from the focal animal. For approaches and departures, we recorded the identity of the partner and the individual responsible for the move into or out of proximity. For social interactions, observers recorded the type of social behavior, the identity of the partner, and whether the interaction was initiated by the focal animal, the partner, or jointly. For vocalizations, observers recorded the type of call given, the identity of the partner, and which individual produced the call.

All data were collected on hand-held computers in the field and later transferred onto computers for error-checking and storage.

### Dominance rank

Assessment of female dominance rank was based on the outcome of aggressive interactions (threats, chases, attacks, submission) and supplants. We used the likelihood-based Elo-rating method [47] to assign ranks to females. The modeling approach implements a maximum likelihood fitting of individuals’ initial Elo-scores when entering the hierarchy and maximum likelihood fitting of the constant *k* which, multiplied by the winning probability of the loser prior to the interaction, determines the increase in Elo-score for the winner and the corresponding decrease in Elo-score for the loser following the interaction. For females in PHG, we analyzed data recorded between January 2013 and December 2016; for females in ENK, we analyzed data recorded between November 2013 and December 2016. *K* was _~_ 0 for both groups, indicating that dominance hierarchies were stable over the study period. Prediction accuracy was 92% for PHG and 82% for ENK which indicates that in most cases the difference in females’ current Elo-scores predicted the outcome of their interaction. Elo-ranks were used to determine whether females were approached by females who ranked higher or lower than themselves.

### Maternal kinship

Maternal kinship relationships among natal females were known from genealogical records extending back to the early 1970’s. The female who immigrated into PHG as a juvenile was assumed to be unrelated to any group members except her own progeny. In the analyses, we compared mother-daughter dyads to all others.

#### Analysis

For each approach, we extracted information about the time of the approach, the identity of the female who initiated the approach (the actor), the identity of the female who was the recipient of the approach (partner), all subsequent vocalizations and social interactions involving the actor and its partner, and the time the bout ended. To assess the length of proximity bouts, we extracted information about the time each proximity bout began and ended, and calculated the difference. Proximity bouts that were ongoing at the end of focal samples were not included in analyses of factors that influenced bout length, grooming solicitations, lip smacks, embraces, and nonaggressive contact).

Because were interested in the impact of grunts on subsequent interactions and the factors that predicted whether females would grunt or remain silent after they approached others, we evaluated the first behavioral event in each approach sequence. We categorized approach sequences that began with grunts from the actor to the recipient as “vocal approaches” and we categorized all other approaches as “silent approaches”. We examined the impact approach type (vocal/silent) on the next interaction in the approach sequence. Following [34] we limited the analyses of the impact of approach type to cases in which the second interaction in the approach sequence occurred within 30 seconds of the approach itself.

We constructed multi-level mixed effect logistic regression models to assess the impact of approach sequence type (vocal = 1, silent = 0) on subsequent interactions initiated by the actor. We examined the effects of sequence type on the likelihood that (a) the actor would behave aggressively or the recipient would behave submissively, (b) the actor would handle the recipient’s infant, and (c) the actor would initiate affiliative interactions with the recipient. We also examined the effects of sequence type on the likelihoods that the recipient would initiate aggression or induce submission by the actor, the recipient would handle the actor’s infant, and the recipient would initiate affiliation to the actor. In each of these models, the outcome variable is coded as 1 if the specified behavior occurred and 0 if it did not occur. Previous analyses of female-female and female-male interactions show that most of the variation in behavior is at the level of the dyad, not the individual [42], so we treated the dyad as a random effects variable in these analyses.

We used multi-level mixed effect linear regression models to assess how sequence type influenced the duration of proximity bouts.

We constructed multi-level mixed effect logistic regression models to assess the factors that affected sequence type. In these analyses, the sequence type is the outcome variable. For models in which we controlled for the effects of additional variables (such as dominance rank or the presences of infants), these variables were treated as fixed effects variables.

The analyses are based on data derived from 6720 15-minute focal samples on 32 adult and subadult females (210.00 ± 14.80 focal samples per female). The sample of approach sequences derived from this dataset includes 16830 approaches involving 822 pairs of females (20.47 ± 0.89 approaches per dyad). Analyses of the impact of approach type (silent/vocal) are based on 8487 cases in which the second interaction in the approach sequence occurred within 30 seconds of the initial approach.

All statistical analyses were conducted with STATA 11. Where appropriate, we report the sample means and standard errors. Data are available from the Dryad Digital Repository: https://doi.org/10.5061/dryad.2vd835g.

### Ethical note

The study conformed to U.S. and Kenyan laws and was approved by the National Commission for Science and Technology of Kenya and the Kenya Wildlife Service. The project was approved by the Arizona State University Institutional Care and Use Committee.

## Results

Approximately 12% of approach sequences began with grunts by the actor (0.12 ± 0.01, n = 822 dyads), and these grunts typically occurred within the first five seconds after the approach (average time elapsed: 5.35 ± 0.63).

### Impact of grunts on subsequent interactions

Vocal approaches were associated with a 74% reduction in the likelihood that actors would behave aggressively toward recipients or induce submission in recipients (Odds ratio (OR): 0.2720 ± 0.0634, z = -5.58, p = 0.001). Vocal approaches were also associated with a 66% reduction in the likelihood that recipients would behave aggressively toward actors or induce submission in them (OR: 0.3468 ± 0.0676, z = —5.43, p < 0.001). In both of these models, we controlled for the effects of the effects of the relative rank of the actor and recipient.

Vocal approaches increased the likelihood of infant handling. When females approached other females with infants under the age of 6 months, grunts increased the likelihood of handling the recipient’s infant 7-fold (OR = 7.5106 ± 0.7860, z = 19.27, p < 0.001). Vocal approaches did not have a significant impact on the likelihood that their own infants would be handled by recipients (OR: 0.8146 ± 0.1825, z = —0.92, p < 0.360).

Vocal approaches also increased the likelihood that actors and recipients would behave affiliatively toward one another, although the magnitude of the effect on affiliation was lower than the magnitude of the effect on infant handling. Vocal approaches increased the likelihood that actors would initiate affiliative behavior toward recipients (OR: 1.4312 ± 0.1403, z = 3.66, p < 0.001). In addition, after vocal approaches, recipients were more likely to initiate affiliative behavior toward actors (1.5865 ± 0.1527, z = 4.79, p < 0.001). In these models, we controlled for the effects of genetic relatedness.

Vocal approaches were associated with longer proximity bouts. Proximity bouts that began with vocal approaches lasted about 22% longer than proximity bouts that began with silent approaches (vocal: 38.14 ± 1.68 seconds, silent: = 31.36 ± 0.63 seconds; multi-level mixed effect linear regression: β = 6.8063 ± 1.6870, z = 4.03, p < 0.001, n = 15092 approach sequences with known endings).

### Predictors of grunting

The presence of infants under the age of 6 months, relative rank, and maternal kinship all influenced the probability that females would grunt as they approached other females. Females were more than four times more likely to grunt when they approached females with young infants than when they approached females who did not have young infants (Multi-level mixed effects logistic regression: Odds ratio: 4.6073 ± 0.2671, z = 26.35, p < 0.001). Females were about 19% more likely to grunt when they approached lower ranking females than when they approached higher ranking females (Odds ratio: 1.1937 ± 0.0579, z = 3.65, p < 0.001), and about 22% less likely to grunt when they approached their own mothers and daughters than when they approached other females (Odds ratio: 0.7854 ± 0.0967 z = -1.96, p = 0.050).

## Discussion

When female olive baboons grunt after they approach other females, they are less likely to behave aggressively toward their partners or intimidate them and less likely to be harassed or intimidated themselves. When females grunt as they approach, they are also more likely to handle their partners’ infants, and more likely to initiate affiliation or receive affiliation from their partners. Females are also likely to remain in proximity longer if they approach and grunt than if they approach and remain silent. Thus, grunts seem to act as reliable signals of benign intent, which are effective in inhibiting aggression from partners, and facilitating nonaggressive social interactions. These results support and extend previous findings derived from observations of chacma baboons [34], and suggest that this is a robust phenomena.

In both chacma and olive baboons, decisions about whether to grunt or remain silent after approaches to other females seem to depend in part on their partners’ likely response to their approach. This is influenced by the presence of infants, partners’ relative rank, and their genetic relationship. In both species, females are substantially more likely to grunt as they approach mothers of young infants than as they approach other females, which suggests that there may be a need for signalers to communicate to mothers of young infants that their intentions are benign. Similarly, females are also more likely to grunt to females who are lower ranking than themselves, and potential victims of aggression, than to those that are higher ranking than themselves and relatively safe from harassment. They are also less likely to grunt to their own mothers and daughters, with whom they maintain close and tolerant social bonds, than they are to grunt to other females.

Although female olive baboons and female chacma baboons seem to be influenced by similar factors when they decide whether to grunt or remain silent, there are some differences in their behavior as well. Overall, the females in our study groups were less likely to grunt after they approached other females than the chacma baboons were (this study: 14% vs 28%, ref. 34). In addition, the magnitude of the odds ratios associated with the predictor variables were all lower for the olive baboons than for the chacma baboons. It is possible these differences reflect subtle variation in the dynamics of social relationships among females in these two species or populations. For example, nepotistic biases in social behavior are less pronounced in our study groups than in chacma baboons or yellow baboons [42], and this might influence females’ decisions about whether to grunt as they approached their relatives. It is also possible that methodological differences may have affected the results. In this study, approaches to within 1 m were recorded while approaches to within 2 m were recorded for the chacma baboons [34].

In summary, female olive and chacma baboons seem to use grunts to signal their intention to behave peacefully. Decisions about whether to call or to remain silent after an approach, are influenced by contextual factors, among which we have identified the relative rank of the two females, whether an infant is present, and genetic relationship between the actor and recipient. The data support the hypothesis that signals of benign intent are particularly important for pairs of females whose relationships are not predictably affiliative and there is uncertainty about the likely outcome of an interaction. Taken together, these data also support the view that baboons and other primates may “exhibit a flexibility in call usage that is similar to the flexibility that they display when responding to the calls of others.” ([13]:1975). At the same, however, subtle differences in the usage of grunts may reflect differences in the dynamics of social relationships across populations or species. Further research is needed on other species to assess the extent of flexibility in call usage and identify the factors that influence animals’ decisions about whether or not to call.
